# *Pseudomonas aeruginosa* Infection Modulates the Immune Response and Increases Mice Resistance to *Cryptococcus gattii*

**DOI:** 10.3389/fcimb.2022.811474

**Published:** 2022-04-25

**Authors:** Eluzia C. Peres-Emidio, Gustavo J. C. Freitas, Marliete C. Costa, Ludmila Gouveia-Eufrasio, Lívia M. V. Silva, Anderson P. N. Santos, Paulo H. F. Carmo, Camila B. Brito, Raquel D. N. Arifa, Rafael W. Bastos, Noelly Q. Ribeiro, Lorena V. N. Oliveira, Monique F. Silva, Tatiane A. Paixão, Alessandra M. Saliba, Caio T. Fagundes, Daniele G. Souza, Daniel A. Santos

**Affiliations:** ^1^ Departamento de Microbiologia/Laboratorio de Micologia, Universidade Federal de Minas Gerais, Belo Horizonte, Brazil; ^2^ Departamento de Microbiologia/Laboratorio de Interação Microorganismo-Hospedeiro, Universidade Federal de Minas Gerais, Belo Horizonte, Brazil; ^3^ Faculdade de Ciencias Farmaceuticas de Ribeirão Preto, Universidade de São Paulo, Ribeirão Preto, Brazil; ^4^ Centro de Biociencias, Universidade Federal do Rio Grande do Norte, Natal, Brazil; ^5^ Departamento de Patologia/Laboratorio de Patologia Celular e Molecular, Universidade Federal de Minas Gerais, Belo Horizonte, Brazil; ^6^ Departamento de Microbiologia e Imunologia, Universidade do Estado do Rio de Janeiro, Rio de Janeiro, Brazil

**Keywords:** Cryptococcosis, coinfection, *Cryptococcus gattii*, *Pseudomonas aeruginosa*, iNOS

## Abstract

Cryptococcosis is an invasive mycosis caused by *Cryptococcus* spp. that affects the lungs and the central nervous system (CNS). Due to the severity of the disease, it may occur concomitantly with other pathogens, as a coinfection. *Pseudomonas aeruginosa* (Pa), an opportunistic pathogen, can also cause pneumonia. In this work, we studied the interaction of *C. gattii* (Cg) and Pa, both *in vitro* and *in vivo*. Pa reduced growth of Cg by the secretion of inhibitory molecules *in vitro*. Macrophages previously stimulated with Pa presented increased fungicidal activity. *In vivo*, previous Pa infection reduced morbidity and delayed the lethality due to cryptococcosis. This phenotype was correlated with the decreased fungal burden in the lungs and brain, showing a delay of Cg translocation to the CNS. Also, there was increased production of IL-1β, CXCL-1, and IL-10, together with the influx of iNOS-positive macrophages and neutrophils to the lungs. Altogether, Pa turned the lung into a hostile environment to the growth of a secondary pathogen, making it difficult for the fungus to translocate to the CNS. Further, iNOS inhibition reverted the Pa protective phenotype, suggesting its important role in the coinfection. Altogether, the primary Pa infection leads to balanced pro-inflammatory and anti-inflammatory responses during Cg infection. This response provided better control of cryptococcosis and was decisive for the mild evolution of the disease and prolonged survival of coinfected mice in a mechanism dependent on iNOS.

## Introduction

Cryptococcosis is an invasive mycosis caused by environmental capsulated yeasts of the genus *Cryptococcus*. It primarily affects the lungs and can reach the central nervous system (CNS), causing meningoencephalitis (Chen et al., 2014; May et al., 2016). Neurocriptococcosis has become an essential concern in the face of the HIV pandemic, presenting high mortality rates (Crump et al., 2011; Quian et al., 2012; Rohatgi and Pirofski, 2015; Skalski and Limper, 2016). Despite coinfection between HIV and *Cryptococcus* spp. being widely studied, cryptococcosis can occur concurrently with other pathogens. Thus, it increases the possibility of multiple interactions with other microorganisms and even with the host microbiota, which may influence the mechanisms of pathogenesis (Nadaud et al., 2007; Dhamgaye et al., 2015; Saleem et al., 2015; Rodrigues, 2018). Coinfections of *C. neoformans* with *Toxoplasma gondii* (toxoplasmosis), *Taenia solium* (neurocysticercosis), *Plasmodium* spp. (malaria), *Prototheca* spp. (protothecosis), *Mycobacterium tuberculosis* (tuberculosis), Influenza virus, and bacteria, like *Streptococcus pneumonie* (bacterial pneumonia), have already been reported (John and Coovadia, 1998; Nadaud et al., 2007; Martinez-Longoria et al., 2015; Saleem et al., 2015; Huang et al., 2019).

*Pseudomonas aeruginosa* (Pa), similarly to *Cryptococcus* spp., inhabits the environment and acts as a human opportunistic pathogen. It infects many organs and tissues, being an important causative agent of nosocomial pneumonia, with about 90,000 deaths yearly (Mulcahy et al., 2014). In addition, Pa is responsible for about 13-23% of nosocomial infections in patients in intensive care units (Driscoll et al., 2007) and has been one of the most common pathogens associated with lung infections in cystic fibrosis patients (Mayer-Hamblett et al., 2014).

Interestingly, microorganisms that share niches, especially bacteria, can develop antagonistic mechanisms against pathogenic fungi. For example, *in vitro* experiments have shown that Pa secretes a massive arsenal of toxins important to pathogenicity. It inhibits the growth of Gram-positive bacteria and fungi, such as *Saccharomyces cerevisiae*, *Candida* spp., *Aspergillus fumigatus*, *Aspergillus niger*, *Fusarium oxysporum*, and *Cryptococcus* spp. (Rabin and Hauser, 2003; Rella et al., 2012; Tupe et al., 2014; Bora, 2016). In addition, Pa induces a robust inflammatory response, which may play an essential role in modulating innate and adaptive immunity (Iwasaki and Medzhitov, 2010; Williams et al., 2010). Thus, since Pa and *Cryptococcus* can cause pnemonia, we hypothesized that Pa could interact with *Cryptococcus* spp. in a bacterial-fungal way and modify the host’s response to the mycosis.

This study investigated the interaction between Pa and *Cryptococcus gattii* (Cg) *in vitro* and *in vivo*. Briefly, our results show that Pa infection reduced mice mortality due to secondary infection by Cg. In addition, the inflammatory mechanisms were addressed as well. Understanding the interaction mechanisms between Cg and Pa can provide mechanisms of pathogenesis and contribute to the development of new drugs or immunotherapies.

## Materials and Methods

### Strains

We used the strains *C. gattii* L27/01 (VGI), *C. gattii* R265 (VGII), *C. neoformans* H99 (VNI) and *P. aeruginosa* ATCC27853 (Manassas, VA, USA). Cg L27/01 and Pa ATCC27853 were tested both *in vitro* and *in vivo*. Other Pa strains were tested *in vitro*: PAO1, PAK, PA103 and PA14. Also, we tested genetically modified strains *in vitro*: PAK*ΔexoS*, Pa103*ΔexoU*, PAO1*ΔH1*, PAO1*ΔH2*, PAO1*ΔH3*, PAO1*ΔH1ΔH2*, PAO1*ΔH1ΔH3*, PAO1*ΔH2ΔH3*, PAO1*ΔH1ΔH2ΔH3*, PA14*ΔpscN*, PA14*ΔpscNΔH2*, PA14*ΔpscNΔvgrG14rhs14*, PA14*ΔpvdA*, PA14*ΔpqsE*, PA14*ΔphZA1* (Saliba et al., 2005; Liberati et al., 2006; Mikkelsen et al., 2011; Pernet et al., 2014; Halder et al., 2019; Wettstadt et al., 2019). All the strains were maintained at -80°C.

### *In Vitro* Assays

*P. aeruginosa* strains were cultured on Cetrimide agar (Acumedia - Neogen Corporation, Michigan, USA) for 24h at 37°C. Then colonies were inoculated in non-pyrogenic saline to obtain an absorbance of 1,040 at 600 nm. Further, it was centrifuged at 8000 rpm for 5 minutes, the supernatant was discarded, and the pellet was resuspended. The volume of 5 μL was used, equivalent to 10^6^ colony forming units (CFU). The *Cryptococcus* strains were cultured on Sabouraud dextrose agar (SDA) (BD Difco™, Heidelberg, Germany). The colonies were suspended in PBS to obtain a 75-77% transmittance at 530 nm, containing approximately 1x10^6^ to 5x10^6^ CFU/mL. In addition, the concentration of the inoculum was adjusted so that each 40 µL contained about 10^4^ CFU.

#### Spot-on-the-Lawn Assay

The spot-on-the-lawn assay was performed as described previously (Harris et al., 1989). Briefly, 5 µL of the Pa inoculum were cultured on brain heart infusion agar (BHI) (Acumedia - Neogen Corporation, Michigan, USA) and incubated at 37°C for 18h. Chloroform was placed on the lids of each plate for 30 min to inactivate the cells. The plates were then uncovered until total chloroform evaporation. Then, the medium was overlaid with 4 mL of BHI soft agar (0.75% agar) inoculated with 1x10^5^ CFU of *Cryptococcus* spp. The plates were incubated for 48h at 37°C, and the reading was performed by visualizing halos of fungal growth inhibition. However, for the evaluation of PAK, PA103, their respectives mutants (PAK*ΔexoS* and Pa103*ΔesxoU*) (Saliba et al., 2005), PAO1 and PA14 and their respectives T6SS and T3SS mutants strains, including double and triple mutants (PAO1*ΔH1*, PAO1*ΔH2*, PAO1*ΔH1ΔH2*, PAO1*ΔH1ΔH2*, PAO1*ΔH1ΔH3*, PAO1*ΔH2ΔH3*, PAO1*ΔH1ΔH2ΔH3*, PA14*ΔpscN*, PA14*ΔpscNΔH2*, PA14*ΔpscNΔvgrG14rhs14*) (Mikkelsen et al., 2011; Wettstadt et al., 2019), PA14 and its mutants for pyocyanin (PA14*ΔphZA1*) and pyoverdine (PA14*ΔpvdA*, PA14*ΔpqsE*) (Liberati et al., 2006) were also evaluated. 5 mM ethylene glycol-bis (b-aminoethylether) N, N, N ′, N ′- tetraacetic acid (EGTA) (Sigma-Aldrich, Missouri, USA) and 20 mM MgCl_2_ were added to BHI agar (Saliba et al., 2006). Detailed information about the strains tested is listed in **Table 1**.

**Table 1:** Strains used in this study.

Parental strain	Mutant strains	Deletion	References
PAK	PAK*ΔexoS*	T3SS (Exo S)	(Saliba et al., 2005)
Pa103	Pa103*ΔexoU*	T3SS (Exo U)	(Saliba et al., 2005)
PAO1	PAO1*ΔH1* PAO1*ΔH2* PAO1*ΔH3* PAO1*ΔH1ΔH2* PAO1*ΔH1ΔH3* PAO1*ΔH2ΔH3* PAO1*ΔH1ΔH2ΔH3*	Three groups of genes: T6SS (H1, H2 e H3)	(Mikkelsen et al., 2011; Wettstadt et al., 2019)
PA14	PA14*ΔpscN* PA14*ΔpscNΔH2* PA14*ΔpscNΔvgrG14rhs14*	T6SS: Rhs (gen H2), Vgr (protein piercing device of T6SS); T3SS: Pscn (ATPase)	(Mikkelsen et al., 2011; Wettstadt et al., 2019)
PA14	PA14*ΔphZA1*	Pyocyanin	(Liberati et al., 2006)
	PA14*ΔpvdA* PA14*ΔpqsE*	Pyoverdine	

#### Co-Culture of Pa and Cryptococcus

Initially, Cg L27/01 (10^5^ CFU) and Pa ATCC27853 were co-cultured with different Pa (10^5^, 10^3^, and 10^2^ CFU) inocula in 96-well plates containing 200 µL of BHI broth, at 37°C. After 1h, 4h, and 12h, aliquots of 50 µL of the co-cultures were plated on SDA containing 0.2 g/L of chloramphenicol to quantify the fungal colonies. After choosing the best Pa inoculum, co-cultures of L27/01, R265, and H99 strains were performed with each of the strains of Pa (ATCC27853, PAK, PA103, PAO1) using 10^5^ CFU of Pa and 10^5^ CFU of fungi. The 96-well plates were incubated for 4 hours at 37°C, and the fungal colonies were further counted on SDA.

#### *In Vitro* Susceptibility Test Cryptococcus spp. to Pyocyanin

The susceptibility of L27/01, R265, and H99 to Pyocyanin (Sigma-Aldrich) was determined by the broth microdilution method according to the M27-A3 guidelines from the Clinical and Laboratory Standards Institute (CLSI, 2008). The pyocyanin minimum inhibitory concentrations (MIC) were considered as 100% of growth inhibition. In addition, 20 μl from each well showing complete inhibition after 72 h were cultured on SDA. The minimum fungicidal concentration (MFC) was the lowest drug concentration that showed no growth. After MIC and MFC test, growth curves of each fungal strain were performed in a subinhibitory concentration (6.4 µg/mL), and the OD (600nm) was determined in 1h intervals during 72h.

#### Phagocytosis Assay

The interaction between Pa and Cg was further tested in a macrophage culture. Briefly, bone marrow cells were harvested from the tibias and femurs of female six-week-old BALB/c mice (Ethical approval protocol n° 77/2018). Then, cells were cultured in bone-marrow-derived macrophages (BMDM) differentiation medium [RPMI (Gibco, California, USA) supplemented with 30% L929 growth-conditioning media, 20% fetal bovine serum (Gibco), 2 mM glutamine (Sigma-Aldrich), 100 units/mL of penicillin-streptomycin (Gibco) and 50 µM of 2-mercaptoethanol (Gibco)] for 1 week at 37°C/5% CO_2_ (Weischenfeldt and Porse, 2008). New media were added every 48h. The supernatant was discarded, and the cells were washed with PBS, followed by the addition of 3 mL of 10 mM PBS/EDTA, and incubated on ice for 10 minutes. The cells adhered to the plate, differentiated as macrophages, were resuspended, and transferred to a sterile polypropylene tube. The BMDMs were centrifuged at 200xg/5 min at 4°C and resuspended in 5 mL of RPMI 1640 medium containing 10% BFS, 2 mM glutamine, 25 mM HEPES (Gibco) pH 7.2, 100 units/mL G penicillin, and 5% L929 cell culture supernatant. The cell viability was determined with Trypan Blue (Sigma-Aldrich), followed by plating in 24-well plates for phagocytic index (PI) and intracellular proliferation rate (IPR) determination; or in 96 well plates for reactive oxygen species, peroxynitrite quantification and MTT viability (Costa et al., 2020).

For the phagocytosis assay, 1x10^5^ macrophages/well were cultured in 24-well plates (containing glass coverslips) in RPMI medium at 37°C, 5% CO_2_ for 24h. Then, macrophages were infected with Pa and Cg, following two infection strategies: (i) Pa (1x10^3^ CFU) was added and maintained for 30 minutes, followed by washing with PBS and further inoculation with Cg (1x10^5^ CFU); ii) Pa was added 30 minutes before Cg, without washing. Uninfected macrophages, infected only with Cg or infected only with Pa, were used as controls. The plates were then incubated at 37°C, 5% CO_2_, and the wells were washed after 3 or 6h, followed by removing and staining the coverslips with the Panotico Rapido dye (LABORCLIN, MG, Brazil). PI was determined after microscopic analyzes and was expressed as the percentage of internalized Cg. To investigate the IPR, non-internalized yeasts were removed by washing the wells with PBS. Then, macrophages were lysed with sterile water and incubated for 30 min at 37°C. Finally, 50 µL of the lysate were plated on SDA to determine the fungal burden. IPR was expressed as the quotient CFU 6h/CFU 3h (Ma and May, 2009).

To quantify ROS and PRN, 2,7-dichlorofluorescein diacetate 10 mM (DCFH-DA; Invitrogen, Life Technologies, California, USA) and dihydrorhodamine 123 20 mM (DHR 123; Invitrogen, Life Technologies) were used, respectively. After incubation for 6 hours at 37°C, the fluorescence was read at excitation wavelengths of 485 nm and emission of 530nm. The data were expressed as arbitrary fluorescence units ± SE (Soares et al., 2011).

Cell viability was assessed using the 3-(4,5-dimethylthiazol-2-yl)-2,5-diphenyltetrazolium bromide (MTT) colorimetric assay. After incubation of the different groups for 6 hours, the cultures were incubated with 10μl of 5% MTT for 3 h. After incubation, 100μl of dimethyl sulfoxide (DMSO) was added to each well and the plates were kept at room temperature until complete solubilization of the precipitate. Absorbance was measured at 570μm using a spectrophotometer, being directly proportional to the cell viability.

### *In Vivo* Assays

#### Mice

Female BALB/c mice (n=6 mice per group), six-to-eight weeks old, from the Centro de Bioterismo of the Federal University of Minas Gerais, were used. Water, food, and light/dark cycles were provided *ad libitum*. We followed the Brazilian Society of Zootechnics/Brazilian College of Animal Experimentation guidelines (available at http://www.cobea.org.br/) and Federal Law 11,794. In addition, animal studies were approved by the Ethics Committee on Animal Use from the Federal University of Minas Gerais (CEUA/UFMG, protocol n° 77/2018). The animals were anesthetized intraperitoneally with ketamine (80 mg/kg) (Cetamin^®^ Syntec, SP, Brazil) and xylazine (15 mg/kg) (Xilazin^®^ Syntec, SP, Brazil) before experimentation. All experiments were performed at least twice.

#### Microorganisms

Cg L27/01 and Pa ATCC 27853 were used in the *in vivo* experiments.

#### Determination of Bacterial Inoculum for Coinfection

Prior to the coinfection experiments, we determined the non-lethal inoculum of Pa. Groups of mice were intranasally inoculated with different inocula (10^9^, 10^7^, or 10^5^ CFU/20 μL of PBS) and animals were monitored daily for survival and weight variation. After choosing the inoculum (10^5^ CFU/mouse), an experiment was conducted to evaluate the bacterial burden in organs to estimate the ideal time-point for tests of Cg coinfection. Mice were infected with Pa and euthanized at different time-points (1, 3, 6, 10, or 15 days post-infection - dpi) to quantify the bacterial burden in lungs, brain, bronchoalveolar lavage fluid (BALF), blood, liver, spleen, heart, lymph nodes and thymus. The organs were removed, ground in PBS (except for BALF and blood), plated on BHI, and incubated at 37°C. Colonies were counted, and the results were expressed in CFU/g or CFU/mL. A total and differential cell count was also performed in BALF using Panotico Rapido staining.

#### ROS and Peroxynitrite Analysis in BALF

The quantification of ROS and PRN levels in BALF was performed 3 dpi. Briefly, BALF was removed and 1x10^5^ cells were incubated with 2,7-dichlorofluorescein diacetate 10 mM (DCFH-DA; Invitrogen, Life Technologies, California, USA) and dihydrorhodamine 123 20 mM (DHR 123; Invitrogen, Life Technologies). After incubation for 30 minutes at 37°C, the fluorescence was read at excitation wavelengths of 485nm and emission of 530nm. The data were expressed as arbitrary fluorescence units ± SE (Soares et al., 2011).

#### Coinfection Survival and Behavior

The inoculum of 1x10^4^ CFU/30 µL of Cg L27/01 was used in coinfection experiments. After the inoculum preparation, the animals were infected intratracheally under anesthesia (Santos et al., 2014). The coinfection was carried out to test the influence of Pa infection before and after Cg infection. Thus, animals were infected with Cg three days before or after Pa infection (time determined according to the experiments described above). Groups of non-infected (NI) and mono-infected animals (only with Cg or Pa) were included as controls. The animals were monitored daily for survival, weight, and behavior.

Further, to carry out the tests with heat killed bacteria (HK), Pa inoculum was incubated at 60°C in a water bath for one hour, then cooled to room temperature and used to intranasal inoculation in animals. HK inocula were plated on BHI medium, incubated at 37°C for 48 hours, to verify confirm the Pa inactivation (Priebe et al., 2002).

#### Behavioral Analysis

All animals were monitored twice daily for survival and for behavior analysis by using the SmithKline/Harwell/Imperial College/Royal Hospital/Phenotype Assessment (SHIRPA) protocol. These tests provide reliable information on murine cerebral dysfunction and their general status. Individual parameters evaluated were grouped into five functional categories: neuropsychiatric state, motor behavior, autonomic function, muscle tone and strength, and reflex and sensory function. The score for each functional category was calculated as the total of the evaluated parameters as described previously (Santos et al., 2014). The **Supplementary Table S1** describes all the parameters analyzed in the SHIRPA protocol.

#### Fungal Burden, Differential Leucocytes Counting, and Histopathology

After analyzing survival and behavior, other groups of mice were infected following the same coinfection protocol to assess fungal burden in BALF, lungs, and brain after 1, 10, or 18 days of Cg infection. Differential leucocyte count was also performed in BALF. The animals were euthanized under anesthesia, and the organs and BALF were removed aseptically to quantify the fungal burden. Lungs and brains were weighed and ground in Petri dishes with 1 mL of sterile PBS and using the plunger of 5 mL syringe. Then, they were cultured on SDA and incubated for 48 hours at 37°C. The colonies were counted, and the results were expressed in CFU/g or CFU/mL.

Histological evaluation was performed in an independent experiment to obtain the lungs of animals after 10 days of Cg infection. The lungs were fixed in formalin and embedded in paraffin for histological sections (5µm) and stained with Hematoxicillin-Eosin (HE), followed by microscopic observation.

#### Lung Myeloperoxidase (MPO) and N-acetylglucosaminidase (NAG) Activities and Cytokines and Chemokine Levels

To better understand the prolonged survival of mice previously infected with Pa, we quantified MPO and NAG activities as described previously, providing an indirect measurement of neutrophil and macrophage accumulation in lungs, respectively (Costa et al., 2016). Fragments of lung tissue (100 mg) were homogenized with 1 ml of extraction buffer containing anti-proteases (0.1mM phenylmethylsulfonyl fluoride, 0.1mM benzethonium chloride, 10mM EDTA, and 20 KI aprotinin A, all purchased from Sigma-Aldrich) and 0.05% Tween 20. The concentrations of IL-1β, IFN-γ, IL-17, IL-10, and CXCL-1 were measured by ELISA using commercially available antibodies from DuoSet Kits (R&D Systems, Minnesota, USA) according to the manufacturer’s instructions.

#### Flow Cytometry

The cell profile in BALF from mice infected with Pa, Cg, and Pa+Cg was determined by flow cytometry. Cg and Pa+Cg groups were tested 10 days after the Cg infection. Otherwise, the Pa group was analyzed three days after the infection with the bacteria, which represents the moment when Cg was inoculated in the coinfected group. For this experiment, 1x10^5^ cells/mice were recovered, centrifuged, and incubated at 4°C for 20 min with a mix of fluorochromes conjugates: anti-CD45 mouse (cat 103131 BioLegend clone 30f11), anti-CD11b mouse (cat 562127 BD Pharmingen clone M1/70), Ly6G anti-mouse (cat.127628 Biolegend clone 1A8), F4/80 anti-mouse (cat. 25-4801-82 e-Biosciense clone BM8), anti-mouse CD4, anti-mouse CD3, and anti-mouse MHCII (cat. 107627 Biolegend clone M4/113.15.2). After incubation, the samples were washed with 150µl of PBS containing 0.5% w/v BSA and centrifuged for 10 minutes at 4°C at 400 x*g*. Next, the supernatant was discarded, the pellet was resuspended in 200 µL of correction solution, incubated for 20 min at 4°C, centrifuged, and washed with PBS. Then, the cells were permeabilized with 200 µL of the permeabilization buffer (eBioscience) and incubated at 4°C for 20 min. After that, cells were washed with permeabilization buffer and the non-conjugated rat anti-mouse iNOS antibody (cat 696802 BioLegend clone w160306c). Then, the conjugated anti-mouse IgG antibody (cat.405406 BioLegend clone: poly4054) was added and incubated for 20 min at room temperature. Cells were then washed, fixed with 4% v/v formaldehyde in PBS, analyzed with a FACSCanto II cytometer. Data were analyzed with FlowJo V10 software (Treestar, Oregon, USA).

#### Survival of Coinfected Mice Under Treatment With Nitric Oxide Synthase (iNOS) Inhibitor

After analyzing the flow cytometry results, a new survival curve was performed to assess the effect of treatment with an iNOS inhibitor (Aminoguanidine - Sigma-Aldrich). Aminoguanidine (1 mg/mL) was added to the animals’ drinking water for 20 days starting on the day of the Cg infection. Infected and non-treated groups (NT), as well as NI animals, were used as controls. The animals were monitored daily for survival.

#### Statistical Analysis

Results are shown as means ± SD. All statistical analyses were performed using GraphPad Prism, version 5.00, for Windows (GraphPad Software, San Diego, CA, USA), and results were considered significant at *p* < 0.05. Kaplan–Meier survival curves were plotted and analyzed using the log-rank test. Phagocytosis, IPR assay, ROS, and PRN measurements were analyzed by ANOVA, followed by a Tukey test to compare different groups. Finally, the CFU/g, cytokine, and chemokine levels were analyzed by the nonparametric Friedman test.

## Results

### Pa Inhibits Cryptococcus Growth *In Vitro*

**Figure 1:**
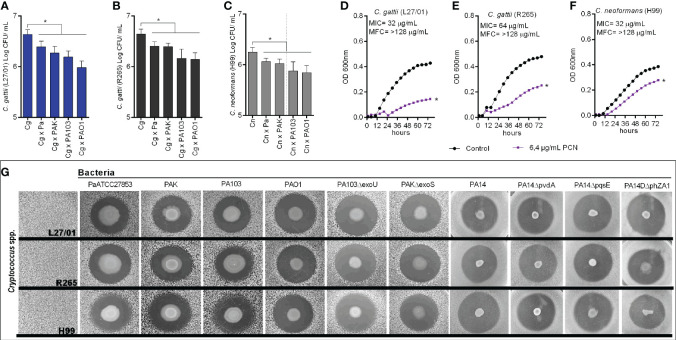
Inhibition of *Cryptococcus* spp. growth by *P. aeruginosa*. **(A–C)** Colony-forming units (CFU) of *Cryptococcus* spp. strains (L27/01, R265, and H99) when co-cultured with different *P. aeruginosa* strains (Pa ATCC27853, PAK, PA103, and PAO1). *p<0.05 when compared with control (fungal culture only). **(D–F)** Growth curves of L27/01, R265, and H99 in the presence of 6.4 µg/mL of pyocyanin (PCN). *p <0.05 compared with control without pyocyanin. MIC: PCN minimum inhibitory concentration. MFC: PCN minimum fungicidal concentration. **(G)** Inhibition of *Cryptococcus* spp. growth obtained from the spot-on-the-lawn technique with Pa (ATCC27853), PAK, PA103, PAO1, PA14 and mutants PAKΔexoS, PA103ΔexoU, PA14*ΔpvdA*, PA14*ΔpqsE*, PA14*ΔphZA1*. Photos show the inhibition zone for each pair of strains tested. Data are representative of three independent experiments.

Initially, we evaluated the *in vitro* effects of Pa (ATCC-27853), PAK, PA103, and PAO1 on the growth of *Cryptococcus* spp. There was a significant reduction in the growth of all strains of *Cryptococcus* (L27/01, R265, and H99) when co-cultured with the bacteria (**Figures 1A–C**).

Pyocyanin MIC was 32 µg/mL for L27/01 and H99, and 64 µg/mL for R265. MFC values ​​were >128 µg/mL for L27/01, R265, and H99 (**Figures 1D–F**). Beyond that, the subinhibitory concentration of pyocyanin (6.4 µg/mL) demonstrated inhibitory effects on fungal growth (**Figures 1D–F**). Further, we observed that the metabolites secreted from the strains of *P. aeruginosa* inhibited the growth of *Cryptococcus* spp., based on the clear and well-defined inhibition halos displayed (**Figure 1G**). In addition, this inhibitory effect was maintained when *P. aeruginosa* mutant strains were tested, including when genes for exotoxin secretion ExoS (PAK*ΔexoS*), ExoU (PA103*ΔexoU*) and pyocyanin (PA14*ΔphZA1*) were deleted (**Figure 1G**). Furthermore, T6SS and T3SS mutant strains, including double and triple mutants, were also evaluated (PAO1*ΔH1*, PAO1*ΔH2*, PAO1*ΔH1ΔH2*, PAO1*ΔH1ΔH2*, PAO1*ΔH1ΔH3*, PAO1*ΔH2ΔH3*, PAO1*ΔH1ΔH2ΔH3*, PA14*ΔpscN*, PA14*ΔpscNΔH2*, PA14*ΔpscNΔvgrG14rhs14*) and all demonstrated the same inhibitory effects (**Supplementary Figure S1**).

### Pa Impairs Cg Phagocytosis, Alters Macrophage’s Oxidative Response, and Reduces Intracellular Proliferation Rate

**Figure 2:**
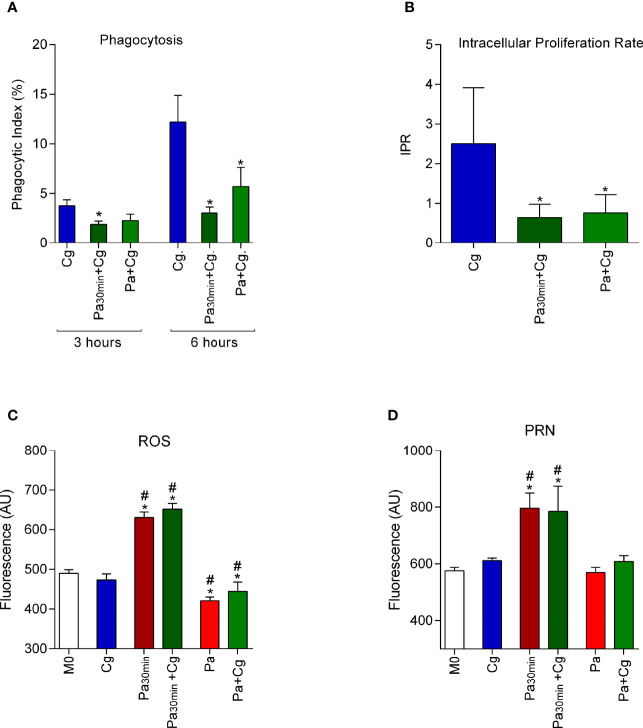
Phagocytic index (PI), intracellular proliferation rate (IPR), and production of reactive oxygen species (ROS) and peroxynitrite (PRN) by bone marrow-derived macrophages (BMDM). Results represent two different protocols: BMDM were stimulated with *P. aeruginosa* for 30 min (Pa_30min_), then washed and infected with *C. gattii (*Cg*)* (Pa_30min_+Cg), or Pa was maintained during all the protocol. **(A)** Phagocytic index after 3h and 6h of the protocol. **(B)** IPR, ratio of 6h/3h CFU obtained in each time point. **(C)** ROS and **(D)** PRN production after 6 hours of infection by *C. gattii*. Cg: BMDM infected only with *C. gattii*; Pa_30min_: BMDM infected with Pa and washed after 30 min; Pa: Pa was maintained during all the protocol; Cg: *C. gattii*; M0: non-infected BMDM. *p <0.05, different from Cg group. #p <0.05 different from M0 group (one-way ANOVA). Data are representative of three independent experiments consisting of six replicates each.

Pa significantly reduced the phagocytic index of Cg, independently of the protocol tested (i.e., 30 min stimulus with Pa and bacterial washing before Cg infection; and BMDM infection with Pa and then Cg with no washing) (**Figure 2A**). In addition, the prior infection with Pa reduced Cg’s ability to grow inside BMDM in both protocols, as attested by the reduced IPR (**Figure 2B**). Interestingly, there was increased production of ROS and PRN when BMDM were stimulated with Pa for 30 min. On the other hand, the presence of Pa during all the protocols led to reduced ROS production and no influence on the PRN levels (**Figures 2C, D**). Macrophages viability, assessed by MTT is presented in **Supplementary Figure S2**.

### Pa Infection Reduces Morbidity and Mortality of Cg-infected Mice

**Figure 3:**
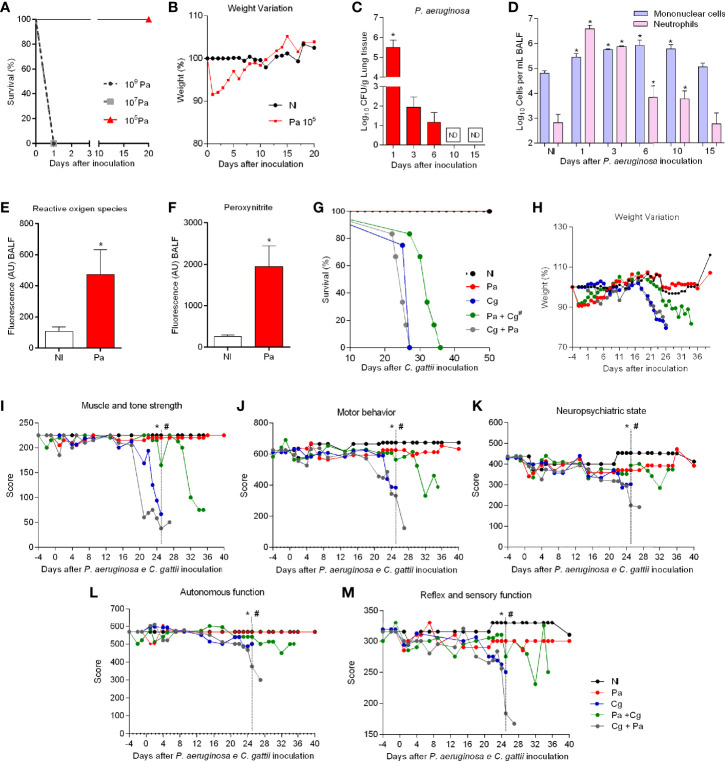
Survival and behavior of mice. **(A)** Six mice per group were inoculated with different inocula of *P. aeruginosa* (Pa) by intranasal infection **(B)** Weight variation after Pa infection with 10^5^ colony forming units (CFU). **(C)** Bacterial burden recovered from the lungs after 1, 3, 6, 10, and 15 days of infection with Pa. *p<0.05. **(D)** Bronchoalveolar lavage fluid (BALF) differential cell counting at different time-points of infection with Pa. *p<0.05. **(E)** Reactive oxygen species (ROS) and **(F)** Peroxynitrite (PRN) levels in (BALF) after three days of infection with Pa. *p<0.05. **(G)** Six mice per group were inoculated with 10^4^ cells of L27/01 strain by intratracheal line inoculation (Cg+Pa - three days before Pa; Pa+Cg - three days after Pa). #p<0.0001 Pa+Cg compared with Cg group (Log-Rank test). **(H)** Weight variation of mice expressed in %. **(I–M)** Five animals per group were submitted to the SHIRPA Protocol. # p <0.05 compared with Cg group (One-Way ANOVA). *p <0.05 compared with the NI group. NI, non-infected; ND, non-detectable. All the experiments were performed at least three times to confirm the data, and the results were always reproducible.

Considering the ability of Pa to inhibit fungal growth both *in vitro* and inside macrophages, we decided to perform tests in an *in vivo* model of coinfection with both pathogens. The first step was to determine the non-lethal Pa inoculum, but able to cause pneumonia. 10^9^ and 10^7^ CFU inocula led to acute and fulminant pneumonia, causing mice death in 24 hours (**Figure 3A**). However, mice infected with the 10^5^ CFU inoculum presented symptoms of pneumonia (piloerection, hyperventilation, back bending, and weight loss), but they have improved after 3 days of infection (**Figures 3A, B**). Mice also lost about 10% of body weight and recovered from the fourth day on (**Figure 3B**). With these results, the 10^5^ CFU inoculum was chosen for the follow-up experiments. Next, we determined bacterial burden in organs to select the ideal time-point to perform coinfection tests. Pa was recovered from the lungs at 1-, 3-, and 6-days post-infection (dpi), with gradual reduction of bacterial burden along with this timeframe and was not detected at 10 and 15 dpi (**Figure 3C**). In addition, Pa was detected in BALF, heart, and thymus only at 1 dpi (data not shown). The cellular recruitment in the BALF changed over the analyzed time points. Neutrophils were predominant at 1 dpi, but their levels were similar to mononucleated cells at 3 dpi. Increased numbers of mononucleated cells were detected at 6- and 10 dpi (**Figure 3D**), with many reactive lymphocytes and spongy alveolar macrophages (data not shown). At 15 dpi the cell profile was similar to the non-infected (NI) mice, with no statistical difference and predominance of epithelial cells, a low number of lymphocytes, and alveolar macrophages. Based on these results, the 3 dpi of Pa was chosen to be the time-point for coinfection experiments. At that time point, there was increased production of ROS and PRN in the BALF (**Figures 3E, F**).

Furthermore, groups of mice were infected with Pa 3 days before (Pa+Cg group) or after (Cg+Pa group) Cg infection. Animals infected only with Cg and Cg+Pa had an average survival of 26 and 25 days, respectively. Pa+Cg infected mice survived longer compared to all the other Cg-infected groups (p <0.0001) (**Figure 3G**). Weight loss occurred earlier in Cg and Cg+Pa groups than Pa+Cg group (**Figure 3H**). In addition to delayed death, mice infected with Pa+Cg presented low morbidity, as attested in all the five parameters evaluated during the SHIRPA protocol (**Figures 3I–M**). Supplementary tables demonstrate mean ± SD for each group tested. Indeed, the protective Pa activity did not occur when we used heat-killed bacteria (**Supplementary Figure S3**).

### Previous Pa Infection Reduces Fungal Burden in BALF, Lungs, and Brain

**Figure 4:**
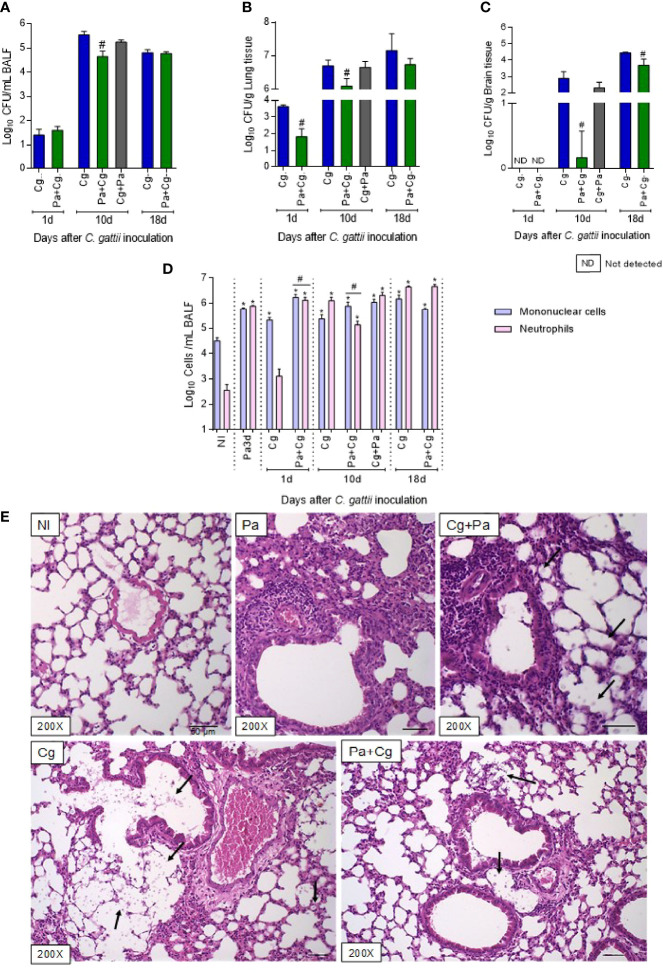
Fungal burden, cell recruitment to bronchoalveolar lavage fluid (BALF), and histopathology. Six mice per group were inoculated with 10^4^ cells of L27/01 strain by intratracheal line inoculation (Cg+Pa - three days before Pa; Pa+Cg - three days after Pa) analyzed at 1, 10 and 18 dpi: Fungal burden in the BALF **(A)**, lungs **(B),** and brain **(C)** of mice infected with *C. gattii* (Cg) or coinfected with *P. aeruginosa.* ^#^p<0.05 compared to Cg group at the same time-point (ANOVA/Tukey’s multiple comparisons test). **(D)** Cell recruitment in the BALF. *p<0.05 compared to the NI group. ^#^p<0.05 compared to the Cg group. **(E)** Histopathological HE staining of the lungs. Representative pictures of the histopathology after ten days of Cg infection; except for the Pa group, which was analyzed 3 dpi of the bacteria infection. Amplification of 200 X. Scale bars of 50 µm. The arrows in Cg and Cg+Pa point to yeasts inside the alveoli, seen as spherical or oval structures, forming clusters, which induce anatomical deformation of the alveoli. The yeasts can also be seen, to a lesser extent, in the group Pa+Cg. NI, non-infected; Cg, *C. gattii*; Pa, *P. aeruginosa*; Cg+Pa, Pa 3 days after infection to Cg; Pa+Cg, Pa 3 days before infection to Cg.

Considering the late mortality of the Pa+Cg group, we next evaluated the fungal burden in the organs, cellular recruitment in the BALF, and lungs’ histology. Even at 1 dpi, a significant reduction of fungal burden was observed in the lungs of coinfected mice, but not in BALF (**Figures 4A, B**). There was no fungal CFUs in the brains for both groups at 1 dpi (**Figure 4C**). Interestingly, the Pa+Cg group demonstrated a lower fungal burden than the Cg group in all the organs analyzed at 10 dpi (**Figures 4A–C**). At this time point, only 40% of mice from the Pa+Cg group had fungi in brain tissue, compared to 100% of the Cg group. After 18 days of Cg infection, the fungal burden of the Pa+Cg group was reduced only in the brain (**Figure 4C**).

The cellular recruitment to the BALF is presented in **Figure 4D**. At the moment of Cg infection (three days after Pa inoculation), mice had increased mononuclear cells and neutrophils compared to the NI group. On the first day after Cg inoculation, the BALF of the Cg group showed an increase in mononucleated cells compared to the NI group. However, the Pa+Cg group showed a significant increase in both cells compared to the NI and the Cg groups. The Pa+Cg group had a predominance of mononucleated cells, while the Cg group showed a predominance of neutrophils at 10 dpi. Although, at 18 dpi, both groups presented increased neutrophils in BALF compared to the NI group (**Figure 4D**).

The histology of the lungs was evaluated after 10 days of Cg infection (**Figure 4E**). Before Cg infection (3 dpi of Pa), lungs showed multifocal and intense inflammatory infiltrate. In the Cg and Cg+Pa groups (this group was included only for histology study), many yeasts were observed in the alveoli and bronchoalveolar branches, in addition to a diffuse and pronounced inflammatory infiltrate. On the other hand, the Pa+Cg group showed a reduction in all parameters analyzed compared to the Cg group, with a lower number of yeasts and slightly multifocal inflammatory infiltrate (**Figure 4E**). **Supplementary Tables S7–S10** demonstrate mean ± SD for each group tested.

### NAG and MPO Activities and Inflammatory Mediators in the Lungs

To evaluate lung inflammation, we quantified NAG and MPO activities and the concentrations of cytokines and chemokines. Tests were performed with organs obtained after 1 and 10 days after fungal infection. Except for the Pa group, which was analyzed after 3 days of bacterial infection alone, representing the time-point of the Cg challenge.

**Figure 5:**
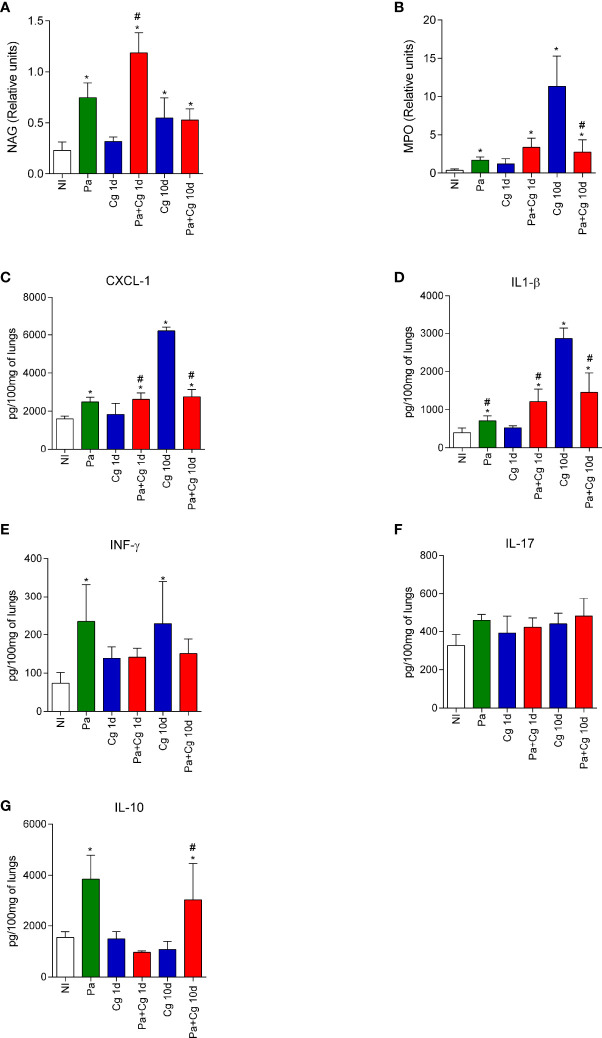
N-acetylglucosaminidase (NAG) and Myeloperoxidase (MPO) activities and cytokines and chemokine levels in the lungs of mice. NAG **(A)** and MPO **(B)** activities. Concentrations of CXCL-1 **(C)**, IL-1β **(D)**, IFN-γ **(E)**, IL-17 **(F)**, and IL-10 **(G)**. *p<0.05 compared with the NI group; ^#^p<0.05 compared with the Cg group at the same time-point (ANOVA/Newman-Keuls multiple comparisons test). Pa: group infected with *P. aeruginosa* and euthanized 3 days after inoculation; Cg: group infected with *C. gattii*; Pa+Cg: group infected with *P. aeruginosa* 3 days before infection with *C. gattii*. Tests were carried out after 1 and 10 days of fungal infection. Data are representative of three independent experiments.

The infection with Pa increased NAG and MPO activities (indicative for macrophages and neutrophils, respectively) and the concentrations of CXCL-1, IL-1β, IFN-γ, and IL-10 compared to the NI group (**Figures 5A-G**). These data represent the inflammatory milieu at the moment of Cg infection in the coinfected mice. The Pa+Cg group presented increased NAG activity and higher levels of CXCL-1 and IL-1β compared to the Cg group at 1 dpi. Coinfected mice analyzed at 10 dpi showed reduced MPO activity, lower concentrations of CXCL-1, IL-1β and IFN-γ, and increased IL-10 levels. No differences were seen when IL-17 was tested (**Figure 5**). **Supplementary Table S11** demonstrates mean ± SD for each group tested.

### Cellular Recruitment in the BALF

**Figure 6:**
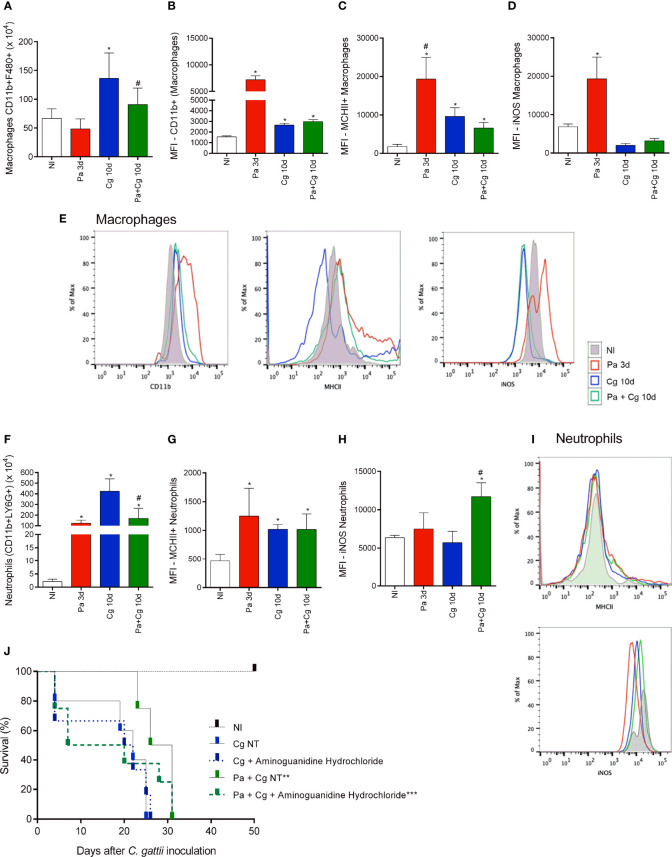
Cellular recruitment to the bronchoalveolar lavage fluid (BALF) of mice and effects of an iNOS inhibitor on the survival curve. **(A)** The number of total macrophages (CD45+ CD11b+ F4/80+); **(B)** total macrophage MFI (CD11b+); **(C)** MFI of activated macrophages (MHCII+) and **(D)** MFI of iNOS+ macrophages; **(E)** Representative histograms of CD11b+ (left), MHC-II (center) and iNOS (right) staining in macrophages (CD45+F4/80+CD11b+ cells) from lung homogenates of mono and co-infected mice; **(F)** Number of total neutrophils (CD11b+ LY6G+); **(G)** MFI of activated neutrophils (MHCII+) and **(H)** MFI of iNOS+ neutrophils; **(I)** Representative histograms of MHC-II (top) and iNOS (bottom) staining in neutrophils CD45+Ly6G+CD11b+ cells) from BALF of mono and coinfected mice. *p<0.05 compared with the NI group. ^#^p<0.05 compared with the Cg group. (ANOVA/Newman-Keuls multiple comparisons test). NI: non-infected group; Pa3d: group infected with *P. aeruginosa* and euthanized 3 days after; Cg: group infected with *C. gattii*; Pa+Cg: group infected with *P. aeruginosa* 3 days before *C. gattii* infection. Cg and Pa+Cg groups were euthanized 10 days after Cg infection. MFI (Median Fluorescence Intensity). **(J)** Survival curve of mice treated and non-treated (NT) with Aminoguanidine Hydrochloride. *** p <0.001 compared with all the other groups. ** p <0.01 compared with the CgNT group (Log-rank test). Flow cytometry data are representative of three independent experiments.

The flow cytometry analysis was carried out using BALF cells from the Pa group (obtained 3 dpi with Pa) and Cg and Pa+Cg groups (10 dpi with the fungus). Data are presented in **Figure 6**. Pa induced the influx of activated macrophages to the BALF, as attested by the subpopulations expressing CD11b and MHCII markers (**Figures 6A–C**). In addition, macrophages present in the lungs of Pa-infected mice presented higher expression of iNOS (nitric oxide synthase on its inducible isoform) (**Figures 6D, E**).

The group infected only with Pa also presented a higher influx of activated neutrophils (**Figures 6F, G**). As seen in macrophages, iNOS labeling was increased in neutrophils in BALF from mice infected only with Pa. Otherwise, iNOS expression remained increased in the Pa+Cg group compared to Cg monoinfected mice (**Figures 6H, I**) **Supplementary Table S12** demonstrates mean ± SD for each group tested.

### Effects of an iNOS Inhibitor on the Survival of Pa+Cg Group

**Figure 7:**
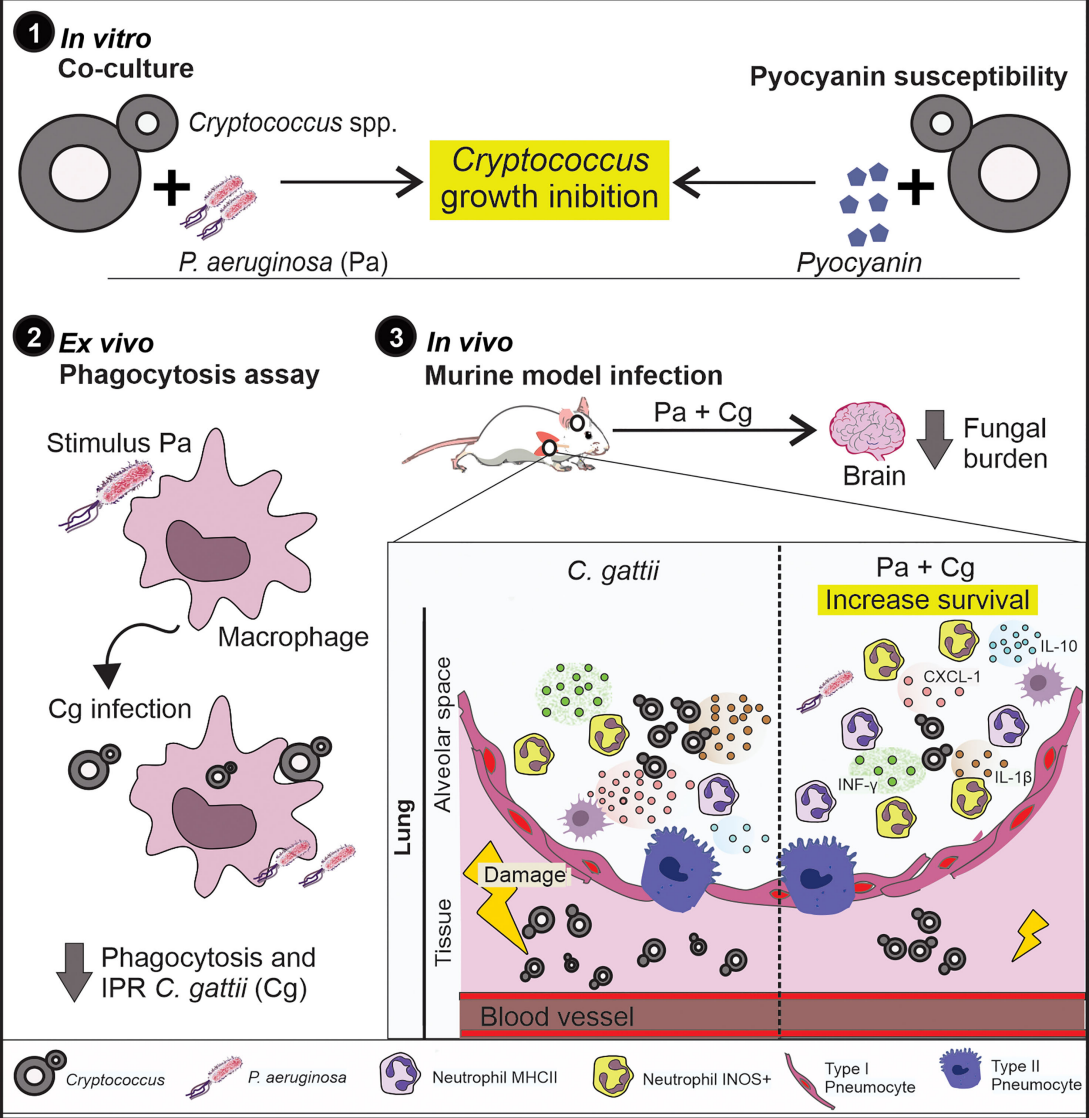
Summary of results. 1) Pa and pyocyanin reduced growth of Cg *in vitro*. 2) Macrophages previously stimulated with Pa presented increased fungicidal activity. 3) *In vivo*, previous Pa infection reduced morbidity and delayed the lethality due to cryptococcosis. This phenotype showed decreased fungal burden in the lungs associated with an increase in IL-1β, CXCL-1 and IL-10 and influx of iNOS positive neutrophils and MHCII neutrophils. Also, we observed a lower fungal burden in the brain at 10 and 18 dpi when mice were previously infected with Pa.

The flow cytometry analysis confirmed the different cell profiles in BALF and pointed to the importance of macrophages and neutrophils expressing iNOS in the Pa+Cg group. To ensure the role of this enzyme in this phenotype, we treated coinfected mice with aminoguanidine hydrochloride, an iNOS inhibitor. The non-treated (NT) Cg and Pa+Cg groups had an average survival of 22 and 28.5 days, respectively. The treated Cg group had a mean survival of 21 days, with no significant differences from the Cg-NT group. Interestingly, the treated Pa+Cg group presented early mortality (average survival = 13.5 days) (**Figure 6J**), providing a reversal (by 60%) of the phenotype. All results from this study are summarized in **Figure 7**.

## Discussion

Cg is an airborne fungal pathogen, which causes pneumonia and meningoencephalitis in immunosuppressed and immunocompetent hosts (Byrnes III et al., 2011; Chen et al., 2014). In this context, the respiratory tract is also a route of Pa infection (Driscoll et al., 2007). Although there is evidence about the inhibitory effect of Pa against *C. neoformans* and *C. gattii in vitro*, it is still unknown how this antagonistic interaction interferes with the progression of cryptococcosis. Microorganisms that share common niches, especially bacteria, may develop antagonistic mechanisms directed against fungi.

The understanding of these interactions may contribute to the development of new drugs or immunotherapies, expanding the therapeutic possibilities and certainly contributing to broadening the understanding of the pathogenesis (Kronstad et al., 2011). Therefore, we carried out this pioneering study to investigate this phenomenon.

Initially, we proved that Pa inhibits Cryptococcus spp. growth during the co-culture and spot-on-the-lawn assays. These data demonstrate that Pa secretes active substances able to kill Cryptococcus spp. T3SS is a host cell-dependent protein secretion pathway and stands out for the secretion of two exotoxins of great invasive power on eukaryotic cells: ExoS and ExoU (Yousefi-Avarvand et al., 2015; Radó et al., 2017). However, even in the absence of these exotoxins, using the strains with deletion for the secretion of ExoS (PAKΔexoS) and ExoU (PA103ΔexoU), other metabolites secreted by these mutants inhibited the growth of C. gattii and C. neoformans (**Figure 1G**). We also analyzed the fungal growth in the presence of different mutant strains with important deletions in T6SS and double/triple mutants for T3SS and T6SS. Similarly, the fungal growth inhibition persisted, pointing that Pa secretes many antifungal metabolites through other secretion systems, such as T2SS, which acts with a variety of important substances, including exotoxin A (Lee et al., 2005). Then we tested the *in vitro* activity of pyocyanin, a vital redox metabolite. Even in sub-inhibitory concentrations, pyocyanin was able to inhibit Cryptococcus spp. growth, as previously demonstrated (Rella et al., 2012). Otherwise, the phenazine-deleted operon strain (PA14ΔphzA1), which is uncapable to produce pyocyanin, was able to inhibit fungal growth. In fact, there are several Pa- produced compounds with antifungal effect against *Aspergillus fumigatus* besides pyocyanin, such as: pyocheline, pyoverdine, dirhamnolipids, and homoserine lactones (Sass et al., 2017; Sass et al., 2019). Altogether, the *in vitro* Pa-Cg interaction is governed by multiple secreted factors, since Pa metabolites may act together in synergism against *C. gattii*. While bacterial metabolites may be important for environmental fungal-bacteria interaction, our macrophage and murine coinfection results suggest that Pa-mediated stimulation of ROS production and iNOS expression also contributes to control of Cg growth by the host.

Based on the *in vitro* results, phagocytosis assays were performed to assess whether the previous contact of Pa with macrophages could influence the Cg phagocytosis. *Cryptococcus* is considered a facultative intracellular pathogen that can survive and replicate in the extracellular environment of alveolar spaces or inside macrophages and neutrophils. The different scenarios after engulfing yeasts show a spectacular ability of this microorganism to manipulate the host’s immune system and use macrophages as an evasion mechanism and to disseminate to the CNS (García-Rodas and Zaragoza, 2012; Sorrell et al., 2016; Wager et al., 2016; Esher et al., 2018). The reduced phagocytosis and IPR of Cg in the presence of Pa points to the probable diminished chances of Cg translocation to the CNS through the “Trojan Horse” mechanism (Sorrell et al., 2016). Interestingly, even when macrophages were able to engulf Cg, the fungicidal activity was more efficient in the presence of Pa. We hypothesized this higher activity is due to increased oxidative and nitrosative bursts when the interaction between Pa and BMDMs occurred for only 30 min. On the other hand, during the continuous exposure to Pa, fungal-bacterial interaction may lead to the reduced Cg burden. In addition, Pa can secrete an arsenal of antioxidant enzymes capable of neutralizing oxidative and nitrosative bursts (Kipnis et al., 2006; Gellatly and Hancock, 2013; Hall et al., 2016), which would explain the reduced levels of ROS and PRN during BMDM continuous exposure to Pa.

These data encouraged us to assess the effects of Pa in the murine model of cryptococcosis. The standardization of Pa infection pointed out that this bacterium induces neutrophils and mononuclear cells recruitment as well as ROS and PRN production in BALF. Therefore, it turned the lung into a hostile environment to the growth of a secondary pathogen, as attested by the results of coinfection experiments. The prior infection with Pa reduced morbidity and delayed lethality due to Cg infection. It was associated with a lower fungal burden in the lungs and a delayed translocation of Cg to the brain. The protective Pa activity did not occur when we used heat-killed bacteria. In this context, we hypothesize that the protective effects induced by Pa may be multifactorial, involving both microorganism-microorganism and microorganism-host interactions. Interestingly, there are evidences that metabolites secreted by Pa can accumulate in different host’s fluids and tissues during infection (Taylor et al., 1995; Caldwell et al., 2009). However, further studies are still needed to clarify if Pa metabolites interact with fungal pathogens during a secondary infection.

Considering the microorganism-host interaction, Pa infection modified the mice response to Cg. Added to the secreted Pa metabolites, it also favours the inhospitable environment for Cg, explaining the reduction in fungal burden right after the first day of infection. Comparing to the Cg-monoinfected mice, the Pa+Cg group presented an increased cellular recruitment and production of inflammatory mediators. The increased number of neutrophils, as well as the levels of CXCL-1, and IL-1β levels during the early stages of Cg infection were associated with the low fungal burden in the lungs and CNS. These mediators were previously related to the resistance to *C. neoformans* infection (Guillot et al., 2008). Interestingly, the response induced by Pa also led to the increased production of IL-10 in the Pa+Cg group during later stages of the infection, which was not observed in the Cg-monoinfected mice. It is possible that this cytokine may have contributed to the modulation of the inflammatory response in coinfected groups. On the other hand, higher levels of proinflammatory mediators in the Cg-monoinfected animals (CXCL-1, IL-1β, and IFN-γ), with concomitantly lower concentrations of IL-10, may have favoured a harmful host response.

Besides modulation of cytokines and chemokines, Pa infection increased iNOS expression in macrophages *in vivo*. iNOS synthesizes nitric oxide (NO), which can be converted to PRN, a powerful oxidizing agent (Licinio et al., 1999) with antimicrobial activity. A previous study (Ghosn et al., 2006) found that treatment with an iNOS inhibitor *in vitro* reduces the internalization of *Cryptococcus*, production of nitric oxide and the fungicidal capacity of macrophages. We demonstrated that iNOS is involved in the interaction between Cg and Pa. However, more studies are needed to better understand the role of this enzyme during coinfection. In addition, there were high levels of neutrophils MHCII+ and iNOS+ in the Pa+Cg group at the late stage of infection. The role of this enzyme in the protection of coinfected mice was further confirmed when we used an iNOS inhibitor, which reduced the survival of Pa+Cg group and partially reversed the protective phenotype. Previous studies demonstrated that, although iNOS knockout mice present adequate production inflammatory mediators, they are more susceptible to *C. neoformans* infection (Aguirre and Gibson, 2000). Furthermore, it was demonstrated that IL-1β regulates the transcription of iNOS and increases expression of this enzyme (Wong et al., 1996; Veluthakal et al., 2006), corroborating our data of the increased IL-1β. Interestingly, previous studies have already shown that iNOS inhibition can also inhibit ROS production (Inoue et al., 2005; Zhao et al., 2010; Forrester et al., 2018). In this context, we hypothesize that iNOS inhibition reduces ROS levels, making the lung less hostile to the fungus, explaining the reduced survival of coinfected mice. Moreover, it would be interesting to perform further experiments to the better characterization of the inflammatory mediators at later time-points (i.e. 10 days post infection) to clarify the persistent changes that may reflect a protective lung response against Cg.

Aminoguanidine treatment was started after 3 days of infection with Pa. At this stage, the animals already showed improvement in the symptoms of Pa infection. Therefore, we believe that treatment with iNOS would most likely not change the survival profile of infection with Pa alone. However, further studies to unequivocally demonstrate the role of iNOS in this interaction and to demonstrate aminoguanidine does not allow reactivation of Pa infection. It is possible that this dose of Pa may become lethal in the absence of iNOS, and hence the mortality is Cg independent. This data does not demonstrate a role for iNOS.

In conclusion, the primary Pa infection leads to balanced pro-inflammatory and anti-inflammatory responses during Cg infection. Altogether, this response provided better control of cryptococcosis and was decisive for the mild evolution of the disease and prolonged survival of coinfected mice. This study provides preliminary information regarding Cg and Pa coinfection in mice, and further research is still needed to clarify the mechanisms involved.

## Data Availability Statement

The raw data supporting the conclusions of this article will be made available by the authors, without undue reservation.

## Ethics Statement

The animal study was reviewed and approved by Ethics Committee on Animal Use from the Federal University of Minas Gerais (CEUA/UFMG, protocol n° 77/2018).

## Author Contributions

EE: performed all experiments procedures *in vitro* and *in vivo*, involving animals analysis, immunological assays, flow cytometry, data analysis, and wrote the paper. GF: contributions to design some experiments, performed experimental procedures (*in vivo*/*in vitro*) and wrote the article. MC: Designed some experiments, performed experimental procedures (*in vivo*/*in vitro*), and contributions to data analysis. LG-E; LS; APNS; PC; NR; LO: performed experimental procedures (*in vivo*/*in vitro*) and contributions to results analysis. CB; RA: contributed to experiments procedures of immunological assays. TP; MS: performed the histopathological analysis. AMS: contributed to developing tests with *Pseudomonas aeruginosa*, their expertise area, and discussed the results. RB; CF; DGS: contributed to the analysis of inflammatory factors and theoretical aspects of immunology of the paper. DAS: coordinated the project, designed and supervised the study, and wrote the article. All authors contributed to the article and approved the submitted version.

## Funding

This study was supported by Fundação de Amparo a Pesquisa do Estado de Minas Gerais - FAPEMIG (PPM-00061-18) and Conselho Nacional de Desenvolvimento Científico e Tecnológico - CNPq (402200/2021-7) and Brazilian Ministry of Health (440010/2018-7). DAS (303762/2020-9) is a research fellow of the CNPq.

## Conflict of Interest

The authors declare that the research was conducted in the absence of any commercial or financial relationships that could be construed as a potential conflict of interest.

## Publisher’s Note

All claims expressed in this article are solely those of the authors and do not necessarily represent those of their affiliated organizations, or those of the publisher, the editors and the reviewers. Any product that may be evaluated in this article, or claim that may be made by its manufacturer, is not guaranteed or endorsed by the publisher.
