# Supplementary material for: Pseudomonas aeruginosa Infection Modulates the Immune Response and Increases Mice Resistance to Cryptococcus gattii
Source: Front Cell Infect Microbiol. 2022 Apr 25;12:811474. doi: 10.3389/fcimb.2022.811474 (PMC9083911; doi:10.3389/fcimb.2022.811474)
Supplement: Supplementary file 1 [file DataSheet_1.docx]

**Supplementary Material**


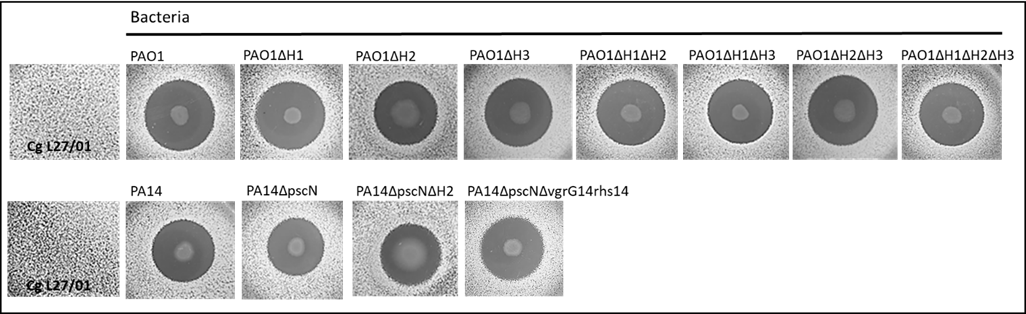


**Supplementary figure S1:** Antagonistic activity of metabolites secreted by different *P. aeruginosa* strains (PAO1 and PA14) and mutants (PAO1ΔH1, PAO1ΔH2, PAO1ΔH3, PAO1ΔH1ΔH2, PAO1ΔH1ΔH3, PAO1ΔH2ΔH3, PAO1ΔH1ΔH2ΔH3, PA14ΔpscN, PA14ΔpscNΔH2, PA14ΔpscNΔvgrG14rhs14) inhibiting *C. gattii* (L27/01 strain) growth.

Supplementary Figure S2: BMDMs Viability assessed through the MTT assay. DMSO: BMDM treated with dimethylsulfoxide. NI: Non-infected BMDM. Pa: BMDM infected with *Pseudomonas aeruginosa* during the whole protocol. Cg: BMDM infected with *Cryptococcus gattii*.

**Supplementary Figure S3:** Survival Curves with heat killed (HK) Pa. **A)** Survival curve of mice infected intratracheally with 10^4^ viables Cg cells or coinfected intranasally with 10^5^ non viables Pa cells (Pa Heat killed (HK)). PaHK: group infected with *P.* *aeruginosa* HK. Cg: group infected with *C. gattii*; PaHK+Cg: group infected with *P. aeruginosa* HK 3 days before infection with *C. gattii*; Cg+PaHK: group infected with *P. aeruginosa* HK 3 days after infection with *C. gattii*. **B)** Survival curve of mice infected intratracheally with 10^4^ viable Cg cells or coinfected intranasally with 10^5^ non viables Pa cells (Pa Heat killed (HK)). PaHK: group infected with *P.* *aeruginosa* HK. Cg: group infected with *C. gattii*; PaHK+Cg: group infected with *P. aeruginosa* HK 5 hours before infection with *C. gattii*.
