# Supplementary material for: Pseudomonas aeruginosa Infection Modulates the Immune Response and Increases Mice Resistance to Cryptococcus gattii
Source: Front Cell Infect Microbiol. 2022 Apr 25;12:811474. doi: 10.3389/fcimb.2022.811474 (PMC9083911; doi:10.3389/fcimb.2022.811474)
Supplement: Supplementary file 2 [file Table_1.docx]

Table S1. SHIRPA (evaluated parameters)

| Functional categories | Parameters (Lackner *et al.*, 2006; Pedroso *et al.*, 2010; Santos *et al.*, 2014; Costa *et al.*, 2016) |
| --- | --- |
| Muscle tone and strength | Grip strength, body tone, limb tone, abdominal tone |
| Motor behavior | Body position, tremor, locomotor activity, pelvic elevation, gait, tail elevation, trunk curl, limb grasping, wire maneuver, negative geotaxis |
| Neuropsychiatric state | Spontaneous activity, transfer arousal, touch escape, positional passivity, biting, fear, irritability, aggression, vocals |
| Autonomous function | Respiration rate, defecation, urination, palpebral closure, piloerection, skin color, heart rate, lacrimation, salivation |
| Reflex and sensory function | Startle response, visual placing, pinna reflex, corneal reflex, toe pinch, righting reflex |

|  | **Group** | | |  |  | | |
| --- | --- | --- | --- | --- | --- | --- | --- |
|  | **NI** | | |  | **Pa 10^5^** | | |
| **DPI** | Mean (%) | SD | N |  | Mean (%) | SD | N |
| 0 | 100 | 0 | 6 |  | 100 | 0 | 6 |
| 3 | 100 | 0 | 6 |  | 93,0 | 3,8 | 6 |
| 20 | 93,0 | 3,8 | 6 |  | 103,8 | 2,3 | 6 |

Table S2: Weight Variation after Pa infection - Mean ± Standard deviation (SD) for each group tested.

|  | **Group** | | |
| --- | --- | --- | --- |
|  | **Pa** | | |
| **DPI** | Mean (CFU) | SD | N |
| 1 | 323750,0 | 136056,0 | 6 |
| 3 | 88,0 | 23,50 | 6 |
| 6 | 14,4 | 9,36 | 6 |
| 10 | 0,0 | 0,00 | 6 |
| 15 | 0,0 | 0,00 | 6 |

Table S3: Bacterial burden recovered from the lungs after 1, 3, 6, 10, and 15 days of infection with Pa - Mean ± Standard deviation (SD) for each group tested.

|  | **Group** | | | | | | | | | | | | | | |
| --- | --- | --- | --- | --- | --- | --- | --- | --- | --- | --- | --- | --- | --- | --- | --- |
|  | **NI ROS** | | |  | **NI PRN** | | |  | **Pa ROS** | | |  | **Pa PRN** | | |
| **DPI** | Mean | SD | N |  | Mean | SD | N |  | Mean | SD | N |  | Mean | SD | N |
| 3 | 107,0 | 57,14 | 5 |  | 262,0 | 64,1 | 5 |  | 473,0 | 274,6 | 5 |  | 1951,0 | 984,7 | 5 |

Table S4: Bronchoalveolar lavage fluid (BALF) differential cell counting at different time-points of infection with Pa - Mean ± Standard deviation (SD) for each group tested.

|  | **Group** | | | | | | | | | | | | | | |
| --- | --- | --- | --- | --- | --- | --- | --- | --- | --- | --- | --- | --- | --- | --- | --- |
|  | **NI Mononuclear cell** | | |  | **NI Neutrophil** | | |  | **Pa Mononuclear cell** | | |  | **Pa Neutrophil** | | |
| **DPI** | Mean | SD | N |  | Mean | SD | N |  | Mean | SD | N |  | Mean | SD | N |
| 1 | 66.000 | 14.309 | 6 |  | 666,7 | 763,8 | 6 |  | 28.3260 | 112.023 | 5 |  | 3.965.000 | 1.574.000 | 5 |
| 3 |  |  |  |  |  |  |  |  | 573.625 | 35.207 | 5 |  | 77.2875 | 47.146 | 5 |
| 6 |  |  |  |  |  |  |  |  | 857.667 | 55.0478 | 5 |  | 6.925 | 13.387 | 5 |
| 10 |  |  |  |  |  |  |  |  | 608.000 | 28.7924 | 5 |  | 6.000 | 6.432 | 5 |
| 15 |  |  |  |  |  |  |  |  | 116.083 | 51.061 | 5 |  | 583 | 1.010 | 5 |

Table S5: Reactive oxygen species (ROS) and Peroxynitrite (PRN) levels in (BALF) after three days of infection with Pa - Mean ± Standard deviation (SD) for each group tested.

|  | **Group** | | |  |  | | |  |  | | |  |  | | |  |  | | |
| --- | --- | --- | --- | --- | --- | --- | --- | --- | --- | --- | --- | --- | --- | --- | --- | --- | --- | --- | --- |
|  | **NI** | | |  | **Pa** | | |  | **Pa+Cg** | | |  | **Cg+Pa** | | |  | **Cg** | | |
| **DPI** | Mean (%) | SD | N |  | Mean (%) | SD | N |  | Mean (%) | SD | N |  | Mean (%) | SD | N |  | Mean | SD | N |
| 24 | 105,5988 | 2,9 | 6 |  | 105,78 | 2,7 | 6 |  | 99,02 | 6,51 | 6 |  | 87,83 | 8,1 | 4 |  | 83,59 | 4,4 | 4 |
| 25 | 98,26958 | 4,0 | 6 |  | 100,08 | 1,0 | 6 |  | 93,20 | 8,04 | 6 |  | 85,19 | 0,8 | 2 |  | 83,11 | 1,6 | 3 |
| 26 | 97,71 | 3,1 | 6 |  | 99,811 | 0,9 | 6 |  | 90,86 | 10,3 | 6 |  | 80,99 | 0,0 | 1 |  | 79,60 | 2,8 | 3 |

Table S6: Weight variation of mice expressed in % - Mean ± Standard deviation (SD) for each group tested.

Table S7: Fungal burden in the bronchoalveolar lavage fluid (BALF) - Mean ± Standard deviation (SD) for each group tested.

| **BALF** | **Groups** | | | | | | | | | | | | | | |
| --- | --- | --- | --- | --- | --- | --- | --- | --- | --- | --- | --- | --- | --- | --- | --- |
|  | **NI** | | |  | **Pa+Cg** | | |  | **Cg+Pa** | | |  | **Cg** | | |
|  |  | | |  |  | | |  |  | | |  |  | | |
| **DPI** | Mean | SD | N |  | Mean | SD | N |  | Mean | SD | N |  | Mean | SD | N |
| 1 | 0 | 0 | 6 |  | 40,00 | 16,33 | 6 |  | - | - | - |  | 25,00 | 19,15 | 6 |
| 10 | 0 | 0 | 6 |  | 45000 | 30000 | 6 |  | 175200 | 40536 | 6 |  | 345000 | 143643 | 6 |
| 18 | 0 | 0 | 6 |  | 58800 | 11628 | 6 |  | - | - | - |  | 63600 | 21882 | 6 |

| **Lungs** | **Groups** | | | | | | | | | | | | | | |
| --- | --- | --- | --- | --- | --- | --- | --- | --- | --- | --- | --- | --- | --- | --- | --- |
|  | **NI** | | |  | **Pa+Cg** | | |  | **Cg+Pa** | | |  | **Cg** | | |
| **DPI** | Mean | SD | N |  | Mean | SD | N |  | Mean | SD | N |  | Mean | SD | N |
| 1 | 0 | 0 | 6 |  | 67,36 | 134,7 | 6 |  | - | - | - |  | 4184 | 1162 | 6 |
| 10 | 0 | 0 | 6 |  | 1260000 | 842442 | 6 |  | 4560000 | 2200000 | 6 |  | 5059000 | 2483000 | 6 |
| 18 | 0 | 0 | 6 |  | 5478000 | 2915000 | 6 |  | - | - | - |  | 14440000 | 30920000 | 6 |

Table S8: Fungal burden in the lungs - Mean ± Standard deviation (SD) for each group tested.

Table S9: Fungal burden in the brain - Mean ± Standard deviation (SD) for each group tested.

| **Brain** | Groups | | | | | | | | | | | | | | |
| --- | --- | --- | --- | --- | --- | --- | --- | --- | --- | --- | --- | --- | --- | --- | --- |
|  | **NI** | | |  | **Pa+Cg** | | |  | **Cg+Pa** | | |  | **Cg** | | |
| **DPI** | Mean | SD | N |  | Mean | SD | N |  | Mean | SD | N |  | Mean | SD | N |
| 1 | 0 | 0 | 6 |  | 0 | 0 | 6 |  | - | - | - |  | 0 | 0 | 6 |
| 10 | 0 | 0 | 6 |  | 1,485 | 2,316 | 6 |  | 196,3 | 246,9 | 6 |  | 764 | 1170 | 6 |
| 18 | 0 | 0 | 6 |  | 4838 | 6554 | 6 |  | - | - | - |  | 27477 | 2806 | 6 |

Table S10: Fungal burden and cell recruitment to bronchoalveolar lavage fluid (BALF) Mean ± Standard deviation (SD) for each group tested.

| **Mononuclear cells** | **NI** | **Pa3d** | **Cg 1d** | **Pa+Cg 1d** | **Cg 10d** | **Pa+Cg 10d** | **Cg+Pa 10d** | **Cg 18d** | **Pa+Cg 18d** |
| --- | --- | --- | --- | --- | --- | --- | --- | --- | --- |
| Mean | 31926 | 596100 | 212400 | 1729000 | 234600 | 764000 | 1099000 | 1496000 | 564080 |
| SD | 10858 | 58782 | 54492 | 556975 | 101954 | 368208 | 382042 | 628876 | 71891 |
|  | | | | | | | | | |
| **Neutrophils** | **NI** | **Pa3d** | **Cg 1d** | **Pa+Cg 1d** | **Cg 10d** | **Pa+Cg 10d** | **Cg+Pa 10d** | **Cg 18d** | **Pa+Cg 18d** |
| Mean | 364,10 | 772875 | 1300 | 1346000 | 1285000 | 136000 | 2077000 | 4464000 | 4638000 |
| SD | 244,40 | 47146 | 1167 | 404565 | 468935 | 54331 | 683398 | 528436 | 950208 |

Table S11: N-acetylglucosaminidase (NAG) and Myeloperoxidase (MPO) activities and cytokines and chemokines levels in the lungs of mice - Mean ± Standard deviation (SD) for each group tested.

| Groups: **(n=6/group)** | | **NI** | **Pa 3d** | **Cg 1d** | **Pa+Cg 1d** | **Cg 10d** | **Pa+Cg 10d** |
| --- | --- | --- | --- | --- | --- | --- | --- |
| **NAG** | Mean | 0,2290 | 0,7466 | 0,3166 | 1,186 | 0,5472 | 0,5286 |
|  | SD | 0,08389 | 0,1451 | 0,04367 | 0,1969 | 0,1967 | 0,1096 |
|  | | | | | | | |
| **MPO** | Mean | 0,3600 | 1,688 | 1,192 | 3,380 | 11,32 | 2,736 |
|  | SD | 0,1813 | 0,4125 | 0,6950 | 1,194 | 3,981 | 1,607 |
|  | | | | | | | |
| **CXCL1** | Mean | 1596 | 2482 | 1833 | 2622 | 6209 | 2760 |
|  | SD | 147,5 | 252,3 | 577,4 | 337,3 | 194,0 | 383,2 |
|  | | | | | | | |
| **IL-1β** | Mean | 397,9 | 703,6 | 525,0 | 1211 | 2865 | 1454 |
|  | SD | 121,6 | 129,9 | 50,10 | 329,6 | 274,3 | 511,2 |
|  | | | | | | | |
| **INF-γ** | Mean | 73,34 | 234,9 | 138,6 | 141,6 | 228,8 | 150,8 |
|  | SD | 28,24 | 97,37 | 29,96 | 23,15 | 111,7 | 37,94 |
|  | | | | | | | |
| **IL-17** | Mean | 326,9 | 458,5 | 392,0 | 421,7 | 439,6 | 481,2 |
|  | SD | 57,42 | 30,49 | 88,53 | 49,48 | 56,28 | 92,37 |
|  | | | | | | | |
| **IL-10** | Mean | 1549 | 3850 | 1495 | 969,2 | 1077 | 3018 |
|  | SD | 221,5 | 937,6 | 283,5 | 55,02 | 318,2 | 1443 |

Table S12: Cellular recruitment to the bronchoalveolar lavage fluid (BALF) of mice - Mean ± Standard deviation (SD) for each group tested.

| Groups: **(n=6/group)** | | **NI** | **Pa 3d** | **Cg 10d** | **Pa+Cg 10d** |
| --- | --- | --- | --- | --- | --- |
| **Macrophages CD11b+F480+ (x 10^4^)** | Mean | 66,69 | 48,49 | 136,2 | 90,68 |
|  | SD | 16,52 | 17,37 | 44,26 | 28,72 |
|  | | | | | |
| **MFI - CD11b+ (Macrophages)** | Mean | 1548 | 7198 | 2660 | 2987 |
|  | SD | 100,6 | 724,0 | 155,5 | 170,5 |
|  | | | | | |
| **MFI - MCHII+ Macrophages** | Mean | 1780 | 21098 | 9619 | 6615 |
|  | SD | 601,7 | 3377 | 2267 | 1448 |
|  | | | | | |
| **MFI - iNOS Macrophages** | Mean | 6855 | 19322 | 2024 | 3191 |
|  | SD | 722,3 | 5635 | 522,8 | 651,5 |
|  | | | | | |
| **Neutrophils (CD11b+LY6G+) (x 10^4^)** | Mean | 2,110 | 123,7 | 424,0 | 169,9 |
|  | SD | 0,9519 | 29,39 | 117,2 | 95,63 |
|  | | | | | |
| **MFI - MCHII+ Neutrophils** | Mean | 463,3 | 1250 | 1020 | 1017 |
|  | SD | 120,6 | 482,2 | 82,10 | 269,7 |
|  | | | | | |
| **MFI - iNOS Neutrophils** | Mean | 6354 | 7496 | 5710 | 11709 |
|  | SD | 319,6 | 2107 | 1487 | 1809 |

REFERENCES

Costa, M. *et al.* (2016) ‘The absence of microbiota delays the inflammatory response to Cryptococcus gattii’, *International Journal of Medical Microbiology*, 306(4), pp. 187–95. doi: 10.1016/j.ijmm.2016.03.010.

Lackner, P. *et al.* (2006) ‘Behavioural and histopathological alterations in mice with cerebral malaria’, *Neuropathology and Applied Neurobiology*, 32(2), pp. 177–188. doi: 10.1111/j.1365-2990.2006.00706.x.

Pedroso, V. S. P. *et al.* (2010) ‘Development of a Murine Model of Neuroparacoccidioidomycosis’, *Journal of Neuroparasitology*, 1, pp. 39–44. doi: 10.4303/jnp/n100402.

Santos, J. R. A. *et al.* (2014) ‘Fluconazole Alters the Polysaccharide Capsule of Cryptococcus gattii and Leads to Distinct Behaviors in Murine Cryptococcosis’, *PLoS ONE*, 9(11), pp. 1–14. doi: 10.1371/journal.pone.0112669.
